# Echocardiographic Predictors of Readmission and Mortality in Elderly Patients With Heart Failure and Preserved Ejection Fraction: A Prospective Observational Study

**DOI:** 10.7759/cureus.92312

**Published:** 2025-09-14

**Authors:** Pradesh Kumar Senthamil Selvan, Pradeepha Rani Pandiyan, Ram Prasanth, Revathi Rajendran, Uma Kiruthika Subramanian Lakshmi, Shahul Irfan

**Affiliations:** 1 Internal Medicine, East Sussex Healthcare National Health Service (NHS) Trust, Eastbourne, GBR; 2 Cardiology, University Hospital Southampton National Health Service (NHS) Foundation Trust, Southampton, GBR; 3 Internal Medicine, Stanley Medical College and Hospital, Chennai, IND; 4 Internal Medicine, Periyar Government Hospital, Perambalur, IND; 5 General Medicine, King's College Hospital, London, GBR

**Keywords:** global longitudinal strain, heart failure with preserved ejection fraction, pasp, resting echocardiography, tapse

## Abstract

Introduction

Heart failure with preserved ejection fraction (HFpEF) is a major cause of hospitalization in the elderly, associated with high morbidity and recurrent admissions despite preserved left ventricular ejection fraction (LVEF ≥50%). Traditional echocardiographic markers may not adequately reflect the underlying myocardial dysfunction. Advanced indices such as global longitudinal strain (GLS), left atrial (LA) reservoir strain, early mitral inflow to early diastolic mitral annular velocity (e/e′) ratio, and the tricuspid annular plane systolic excursion to pulmonary artery systolic pressure (TAPSE/PASP) ratio offer better prognostic insight. This study aimed to evaluate the predictive value of these echocardiographic parameters in determining mortality and heart failure readmissions over a 12-month period in elderly HFpEF patients.

Study

A prospective observational study was conducted at Stanley Medical College, Chennai, from February 2023 to December 2024. A total of 220 patients aged ≥60 years, admitted with acute decompensated HFpEF, were included. Standard and advanced echocardiography were performed within 72 hours of stabilization. Parameters measured included GLS, LA reservoir strain, E/e′ ratio, LA volume index, TAPSE, and PASP. Patients were followed up at 30 days, 90 days, six months, and one year for readmission and mortality outcomes. Statistical significance was set at p<0.05.

Results

The mean age of the patients was 68.4±6.2 years; 126 patients (57.3%) were men. Impaired GLS (< -16%) was found in 138 patients (62.7%), reduced LA strain (< 23%) in 143 (65.0%), elevated E/e′ (>15) in 157 (71.4%), and abnormal TAPSE/PASP (< 0.42) in 81 (36.8%). At 12 months, 129 patients (58.6%) had at least one readmission, and 41 patients (18.6%) had died. GLS < -16% was associated with 91 readmissions (65.9%) and 23 deaths (16.7%) vs. 38 readmissions (46.3%) and 18 deaths (22.0%) in the GLS >-16 group. Patients with LA strain < 23% had 95 readmissions (66.4%) and 25 deaths (17.5%) vs. 34 (44.2%) and 16 (20.8%) patients with LA stain >23%. Elevated E/e′ >15 predicted 103 readmissions (65.6%) and 31 deaths (19.7%) vs. 26 (41.3%) and 10 (15.9%) in those having E/e′ ≤15. TAPSE/PASP <0.42 was linked to 66 readmissions (81.5%) and 19 deaths (23.5%) compared to 63 readmissions (45.3%) and 22 deaths (15.8%) in those with TAPSE/PASP ≥0.42. All associations were statistically significant.

Conclusion

Advanced echocardiographic markers - GLS, LA reservoir strain, E/e′, and TAPSE/PASP - were strong predictors of one-year readmission and mortality in elderly HFpEF patients. These indices offer superior risk stratification compared to conventional parameters and may guide post-discharge planning. Incorporating them into routine inpatient evaluation could improve outcomes through early identification of high-risk individuals and targeted follow-up strategies.

## Introduction

Heart failure with preserved ejection fraction (HFpEF) accounts for about half of all heart-failure presentations in contemporary practice and is particularly concentrated in older adults, especially women, where multimorbidity, vascular stiffening, and exercise intolerance are common [1]. Despite a preserved left-ventricular ejection fraction, these patients experience substantial symptom burden, recurrent congestion, and high utilization of inpatient services, underscoring the syndrome’s clinical and economic weight [1]. Following an index hospitalization, short-term outcomes are sobering in the elderly HFpEF population: approximately one-third of patients are rehospitalized or die within 90 days, reflecting the combined impact of cardiac dysfunction and age-associated comorbidities on recovery and stability [2]. The mechanistic substrate is heterogeneous: diastolic dysfunction, impaired reserve, atrial myopathy, pulmonary vascular disease, and extracardiac factors (e.g., renal dysfunction, sarcopenia, systemic inflammation) each contribute and this heterogeneity helps explain why trial results have been inconsistent and why bedside risk stratification remains challenging [1,2]. Against this backdrop, an inpatient thesis that focuses on identifying robust, reproducible echocardiographic predictors of readmission and mortality in elderly HFpEF can deliver actionable insights for discharge planning and early post-discharge surveillance.

Echocardiography is the pragmatic core of HFpEF evaluation because it noninvasively characterizes chamber remodeling, filling pressures, myocardial mechanics, and right-sided coupling at the bedside. A recent meta-analysis pooling 46 studies and >20,000 HFpEF patients showed three measures that consistently predict cardiovascular death and HF hospitalization across sensitivity analyses: more impaired left-ventricular global longitudinal strain (GLS), lower left-atrial (LA) reservoir strain, and a lower tricuspid annular plane systolic excursion to pulmonary-artery systolic pressure ratio (TAPSE/PASP) [3]. These markers capture complementary biology, subclinical systolic dysfunction, atrial myopathy with elevated filling pressures, and right-ventricular-pulmonary vascular uncoupling, suggesting that a multidimensional echo profile may outperform any single index [3]. Cohort data reinforce this: LA reservoir strain has emerged as a powerful correlate of adverse events in HFpEF, often providing prognostic information beyond traditional volumetric indices and tracking with functional capacity and pulmonary vascular load [4]. Likewise, GLS abnormalities are common in HFpEF and independently associated with composite events and future decline in ejection fraction, supporting its use for risk enrichment in elderly inpatients [5]. Building on this evidence base, the present study prospectively evaluated a standardized panel of advanced echocardiographic parameters, including global longitudinal strain, LA reservoir strain, e/e′ ratio, LA volume index, TAPSE/PASP ratio, RV function, and valvular lesions, while accounting for common cardiometabolic comorbidities such as hypertension and diabetes as potential effect modifiers. This design preserves generalizability to all elderly HFpEF admissions yet quantifies whether common cardiometabolic comorbidities attenuate or amplify the prognostic value of key echo markers, enabling a practical risk model to guide diuretic strategy, follow-up intensity, and targeted referral pathways. The novelty of this study lies in evaluating prognostic echocardiographic markers specifically in elderly inpatients with HFpEF in an Indian population, a group underrepresented in prior meta-analyses and predominantly Western outpatient cohorts.

## Materials and methods

This prospective observational cohort study was conducted in the Department of Cardiology at Stanley Medical College and Hospital, Chennai, over a two-year period from February 2023 to May 2025. Consecutive patients aged 60 years and above who were admitted with acute decompensated heart failure and met the criteria for heart failure with preserved ejection fraction (HFpEF) were included. HFpEF was defined, according to the European Society of Cardiology (ESC) guidelines, as a left ventricular ejection fraction (LVEF) of ≥50% on transthoracic echocardiography, together with objective evidence of diastolic dysfunction or structural heart disease. Diastolic dysfunction was diagnosed if the average e/e′ ratio was >15, or if it was between eight and 15 in the presence of either elevated NT-proBNP, left atrial volume index (LAVI) >34 mL/m², concentric left ventricular remodeling or hypertrophy, or increased tricuspid regurgitation velocity [6]. Patients were excluded if they had an LVEF <50%, an acute coronary syndrome during the index admission, significant primary valvular heart disease as the main cause of heart failure, infiltrative or restrictive cardiomyopathies, severe anemia or thyrotoxicosis as alternative explanations for decompensation, or inadequate echocardiographic image quality precluding core measurements. The sample size was estimated using the formula for proportions, assuming an anticipated prevalence of heart failure of 15%, with 95% confidence level and 5% margin of error, which yielded a minimum required sample size of 196 patients. A total of 220 patients fulfilled the eligibility criteria and were enrolled in the final analysis.

Baseline demographic, anthropometric, and clinical data were collected at admission, including age, sex, body mass index, duration of heart failure symptoms, New York Heart Association (NYHA) class, prior heart failure hospitalizations, and comorbidities such as hypertension, diabetes mellitus, chronic kidney disease, coronary artery disease, atrial fibrillation, chronic obstructive pulmonary disease, and obesity. For consistency, ‘elderly’ was defined as patients aged ≥60 years, reflecting Indian demographic standards and clinical practice. Lifestyle history (smoking, alcohol use) and chronic medication use were documented. On admission, vital parameters (blood pressure, heart rate, respiratory rate, oxygen saturation), clinical signs (jugular venous pressure, pedal edema, pulmonary crepitations), and relevant laboratory values (complete blood count, renal and liver function tests, serum electrolytes, fasting and post-prandial glucose, glycated hemoglobin (HbA1c), lipid profile, and N-terminal pro-B-type natriuretic peptide (NT-proBNP) levels were recorded. NT-proBNP cut-offs were defined according to established age- and sex-adjusted recommendations.

Comprehensive transthoracic echocardiography was performed for all participants within 72 hours of clinical stabilization. All studies were acquired by experienced sonographers and interpreted by cardiologists blinded to patient outcomes. Standard measurements were obtained according to the American Society of Echocardiography guidelines, including LVEF by biplane Simpson method, left ventricular mass index (LVMI), mitral inflow velocities (E, A, E/A ratio), tissue Doppler e′ velocities (septal and lateral) with averaged e/e′, deceleration time, isovolumic relaxation time, LAVI, tricuspid annular plane systolic excursion (TAPSE), right ventricular fractional area change (RVFAC), pulmonary artery systolic pressure (PASP), and grading of valvular lesions. Speckle-tracking echocardiography was used when feasible to obtain GLS and LA reservoir strain. GLS and LA reservoir strain analysis were feasible in over 90% of the patients, while those with suboptimal echocardiographic windows or technically inadequate speckle-tracking images were excluded. We included additional parameters such as left atrial volume index and valvular lesions in accordance with the American Society of Echocardiography (ASE)/European Association of Cardiovascular Imaging (EACVI) recommendations, as these abnormalities are common in elderly HFpEF and contribute to structural remodeling despite weaker prior prognostic evidence. E/e′ was also analyzed alongside GLS, LA strain, and TAPSE/PASP because it remains a guideline-endorsed index of diastolic filling pressures, ensuring comparability with prior HFpEF studies and utility in resource-limited settings. In atrial fibrillation, strain and GLS values were averaged over three cardiac cycles to minimize variability. The cut-off thresholds for these echocardiographic values were adopted from ESC guidelines [6]. Intra- and inter-observer variability was assessed in a random 10% sample.

Patients were followed up at 30 days, 90 days, six months, and 12 months after discharge through outpatient visits or structured telephone interviews. The primary outcomes were all-cause mortality, cardiovascular mortality, and heart failure-related readmission. Secondary outcomes included the composite of mortality or HF readmission, worsening NYHA functional class, and deterioration in renal function. Readmissions were confirmed using hospital records or documentation from other healthcare facilities, while deaths were verified through medical records, family interviews, and, when available, official death certificates. An independent outcome adjudication committee, blinded to echocardiographic findings, reviewed all uncertain events. Data were entered into a secure database with double-entry verification. Continuous variables were expressed as mean±standard deviation. Categorical variables were presented as frequencies and percentages, with comparisons made using the chi-square test or Fisher’s exact test. A p-value <0.05 was considered statistically significant. All statistical analyses were performed using SPSS version v29 (IBM Corp., Armonk, NY).

## Results

Among the 220 elderly patients included in the study, the mean age was 68.4±6.2 years, and the average BMI was 25.6±3.9 kg/m², indicating that most were overweight. There was a slight male predominance, with 126 (57.3%) patients being male. The NYHA classification revealed that the majority were in Class III (118 patients, 53.6%), followed by Class II (54 patients, 24.5%) and Class IV (48 patients, 21.8%). Nearly half of the cohort, 102 patients (46.4%), had experienced at least one prior hospitalization for heart failure, reflecting the chronic relapsing nature of HFpEF in the elderly (Table 1).

**Table 1:** Demographic details of the patients and their NYHA classification at presentation. N: Number of patients; NYHA: New York Heart Association.

Parameter	Mean±SD, N (%)
Age (years)	68.4±6.2
BMI (kg/m²)	25.6±3.9
Sex (Male)	126 (57.3%)
NYHA Class III	118 (53.6%)
NYHA Class II	54 (24.5%)
NYHA Class IV	48 (21.8%)
Previous HF Hospitalizations	102 (46.4%)

Echocardiographic analysis revealed an average LVEF of 58.2%, fulfilling the criteria for HFpEF. However, advanced parameters revealed significant dysfunction: 138 patients (62.7%) showed impaired GLS, suggesting occult systolic dysfunction despite preserved EF. LA reservoir strain was reduced in 143 patients (65.0%), indicating atrial myopathy, while 157 patients (71.4%) had elevated e/e′ ratios, reflecting raised LV filling pressures. Additionally, LAVI was elevated in 172 patients (78.2%), TAPSE was reduced in 65 patients (29.5%), and TAPSE/PASP ratio was abnormal in 81 (36.8%), highlighting right heart dysfunction and pulmonary vascular uncoupling, critical components in HFpEF pathophysiology (Table 2).

**Table 2:** Echocardiographic profile of study population. N, Number of patients; GLS: global longitudinal strain; LVEF: left ventricle ejection fraction; LA reservoir strain: left atrial reservoir strain; LAVI: left atrial volume index; TAPSE: tricuspid annular plane systolic excursion; PASP: pulmonary artery systolic pressure; E/e': early mitral inflow to early diastolic mitral annular velocity ratio

Echocardiographic Parameter	Mean ± SD	Abnormal, N (%)	Normal Range
LVEF (%)	58.2±3.1	-	>50%
GLS (%)	-14.2±2.9	138 (62.7%)	≤ -16%
LA Reservoir Strain (%)	18.3±5.1	143 (65.0%)	≥23%
E/e′ Ratio	16.7±3.4	157 (71.4%)	≤15
LAVI (mL/m²)	42.8±9.3	172 (78.2%)	<34 mL/m²
TAPSE (mm)	17.5±2.8	65 (29.5%)	≥ 17 mm
TAPSE/PASP	0.37±0.09	81 (36.8%)	≥ 0.42

Baseline laboratory parameters revealed significant derangements reflective of underlying cardiac and systemic stress in the HFpEF population. NT-proBNP levels were markedly elevated in 181 patients (82.3%), far exceeding the normal threshold of 125 pg/mL, confirming myocardial strain and volume overload. Anemia was present in 75 individuals (34.1%) with hemoglobin values below 13 g/dL, which may worsen functional status and limit exercise tolerance. Electrolyte imbalances such as hyponatremia were found in 41 patients (18.6%), and 21 patients (9.5%) had abnormal potassium levels, indicating neurohormonal activation and possible diuretic-related effects. Renal dysfunction was also prevalent, with 61 patients (27.7%) having creatinine levels above 1.2 mg/dL. Furthermore, cardiometabolic abnormalities were substantial: 109 patients (49.5%) had poor glycemic control with HbA1c above 5.7%, and 92 patients (41.8%) had low-density lipoprotein (LDL) cholesterol levels exceeding 100 mg/dL (Table 3). These abnormalities collectively highlight the multisystem burden and prognostic implications in elderly HFpEF patients.

**Table 3:** laboratory parameters of study population. NT-proBNP: N-terminal pro-B-type natriuretic peptide; LDL: low-density lipoprotein; HbA1c: glycated hemoglobin.

Laboratory Parameter	Mean ± SD / Median (IQR)	Abnormal -N (%)	Normal Range
NT-proBNP (pg/mL)	2560 (IQR 1800–4800)	181 (82.3%)	<125 pg/mL
Hemoglobin (g/dL)	11.8±1.6	75 (34.1%)	13-17 g/dL
Sodium (mmol/L)	135.4±3.9	41 (18.6%)	135-145 mmol/L
Creatinine (mg/dL)	1.4±0.6	61 (27.7%)	0.7-1.2 mg/dL
HbA1c (%)	7.4±1.2	109 (49.5%)	<5.7%
LDL Cholesterol (mg/dL)	118±32	92 (41.8%)	<100 mg/dL
Potassium (mmol/L)	4.1±0.5	21 (9.5%)	3.5-5.0 mmol/L

Readmission rates rose steadily across all intervals, affecting over half the cohort (58.6%) by one year. The all-cause mortality was 5.0% at 30 days and reached 18.6% at 12 months. Notably, cardiac mortality constituted the majority of deaths: 11.8% by one year compared to 6.8% for non-cardiac causes (Table 4). This breakdown emphasizes that while HFpEF is influenced by comorbidities, a significant proportion of adverse events remain cardiovascular in nature, necessitating cardiology-led follow-up.

**Table 4:** Outcome measure of the study population.

Outcome Measure	30 Days	90 Days	6 Months	1 Year
Readmission	47 (21.4%)	76 (34.5%)	98 (44.5%)	129 (58.6%)
All-Cause Mortality	11 (5.0%)	18 (8.2%)	29 (13.2%)	41 (18.6%)
Cardiac Mortality	7 (3.2%)	12 (5.5%)	19 (8.6%)	26 (11.8%)
Non-Cardiac Mortality	4 (1.8%)	6 (2.7%)	10 (4.5%)	15 (6.8%)

For GLS, patients with impaired GLS (< -16%) had a mortality rate of 16.7% compared to 22.0% in those with preserved GLS (≥ -16%), a difference that was not statistically significant, whereas readmission was significantly higher in the impaired GLS group (65.9% vs. 46.3%). Similarly, patients with reduced LA reservoir strain (< 23%) had comparable mortality to those with preserved strain (17.5% vs. 20.8%) but experienced significantly more readmissions (66.4% vs. 44.2%). In terms of diastolic function, elevated e/e′ (> 15) was not associated with a difference in mortality (19.7% vs. 15.9%) but was linked to a higher rate of readmissions (65.6% vs. 41.3%), reflecting its role in identifying patients with elevated filling pressures prone to recurrent decompensation. The TAPSE/PASP ratio also showed no significant association with mortality (23.5% vs. 15.8%) but demonstrated a strong relationship with readmissions, with 81.5% of patients with a ratio <0.42 being rehospitalized compared to 45.3% with preserved coupling. These findings suggest that while none of the studied echocardiographic parameters independently predicted mortality, impaired GLS, reduced LA strain, elevated e/e′, and abnormal TAPSE/PASP ratio were all strongly associated with increased risk of readmission, highlighting their utility in predicting morbidity rather than mortality in this cohort.

**Table 5:** Prognostic value of echo parameters in study population. p value <0.05 is considered as statistically significant. *p<0.05. LA reservoir strain: left atrial reservoir strain; GLS: global longitudinal strain; TAPSE: tricuspid annular plane systolic excursion; PASP: pulmonary artery systolic pressure; E/e': early mitral inflow to early diastolic mitral annular velocity ratio

Echocardiographic Parameter	Category	n (%)	Mortality n (%)	Chi-square (Mortality)	p-value (Mortality)	Readmission n (%)	Chi-square (Readmission)	p-value (Readmission)
GLS	< -16%	138 (62.7%)	23 (16.7%)	0.6309	0.4270	91 (65.9%)	7.3594	0.0067*
GLS	≥ -16%	82 (37.3%)	18 (22.0%)			38 (46.3%)		
LA Reservoir Strain	<23%	143 (65.0%)	25 (17.5%)	0.1743	0.6764	95 (66.4%)	9.3435	0.0022*
LA Reservoir Strain	≥23%	77 (35.0%)	16 (20.8%)			34 (44.2%)		
E/e′	>15	157 (71.4%)	31 (19.7%)	0.2259	0.6346	103 (65.6%)	9.9971	0.0016*
E/e′	≤15	63 (28.6%)	10 (15.9%)			26 (41.3%)		
TAPSE/PASP	<0.42	81 (36.8%)	19 (23.5%)	1.4937	0.2217	66 (81.5%)	26.1157	0.0001*
TAPSE/PASP	≥0.42	139 (63.2%)	22 (15.8%)			63 (45.3%)		

For GLS, mortality was similar across categories, with no significant difference between severe impairment and normal level (25.0% vs. 22.0%, p=0.8834) or moderate and normal (13.3% vs. 22.0%, p=0.1806). However, readmission was significantly higher in patients with severe GLS impairment compared to normal GLS level (80.0% vs. 46.3%, p=0.0009), while the moderate group showed a non-significant trend toward higher readmissions (60.2% vs. 46.3%, p=0.0876). For LA reservoir strain, mortality did not differ significantly between severe strain and normal level (30.0% vs. 20.8%, p=0.3786) or moderate and normal level (12.6% vs. 20.8%, p=0.2048), but readmission was markedly higher in the severe group (85.0% vs. 44.2%, p=0.0001), whereas the moderate group did not differ significantly from normal (51.5% vs. 44.2%, p=0.4128).

An analysis of e/e′ revealed no mortality difference between severe and normal levels (22.6% vs. 15.9%, p=0.4685) or moderate and normal levels (17.9% vs. 15.9%, p=0.9086), but readmission was significantly increased in both severe (74.2% vs. 41.3%, p=0.0004) and moderate groups (60.0% vs. 41.3%, p=0.0319). TAPSE/PASP <0.32 showed a numerical trend toward higher mortality compared to the normal ratio (32.1% vs. 15.8%, p=0.0785), but this was not statistically significant, while moderate impairment (0.32-0.419) was comparable to normal level (18.9% vs. 15.8%, p=0.7727). Importantly, readmission was significantly higher in both severe (92.9% vs. 45.3%, p<0.0001) and moderate groups (75.5% vs. 45.3%, p=0.0003) (Table 6). Overall, the findings show that severity stratification of these echocardiographic markers did not translate into significant differences in mortality in this cohort, although a borderline trend was seen with severely reduced TAPSE/PASP. In contrast, severe GLS, severe LA strain reduction, elevated e/e′, and impaired TAPSE/PASP were strongly associated with increased risk of readmission, indicating that the burden of diastolic dysfunction and right ventricular-pulmonary coupling abnormalities is more predictive of rehospitalization than mortality in elderly patients with preserved ejection fraction.

**Table 6:** Outcome prediction by severity of echo parameters. A p-value <0.05 is considered as statistically significant. *p <0.05. LA reservoir strain: left atrial reservoir strain; GLS: global longitudinal strain; TAPSE: tricuspid annular plane systolic excursion; PASP: pulmonary artery systolic pressure; E/e': early mitral inflow to early diastolic mitral annular velocity ratio

Echocardiographic Parameter	Severity Category	n (%)	Mortality n (%)	Chi-square (Mortality)	p-value (Mortality)	Readmission n (%)	Chi-square (Readmission)	p-value (Readmission)
GLS	Severe (> –12%)	40 (18.2%)	10 (25.0%)	0.0215	0.8834	32 (80.0%)	11.1161	0.0009*
GLS	Moderate (–12% to –16%)	98 (44.5%)	13 (13.3%)	1.7926	0.1806	59 (60.2%)	2.9173	0.0876
GLS	Normal (≥ –16%)	82 (37.3%)	18 (22.0%)			38 (46.3%)		
LA Reservoir Strain	Severe (< 12%)	40 (18.2%)	12 (30.0%)	0.7751	0.3786	34 (85.0%)	16.4033	0.0001*
LA Reservoir Strain	Moderate (12–22.9%)	103 (46.8%)	13 (12.6%)	1.6080	0.2048	53 (51.5%)	0.6707	0.4128
LA Reservoir Strain	Normal (≥ 23%)	77 (35.0%)	16 (20.8%)			34 (44.2%)		
E/e′	Severe (> 20)	62 (28.2%)	14 (22.6%)	0.5254	0.4685	46 (74.2%)	12.5539	0.0004*
E/e′	Moderate (15–20)	95 (43.2%)	17 (17.9%)	0.0132	0.9086	57 (60.0%)	4.6046	0.0319
E/e′	Normal (≤ 15)	63 (28.6%)	10 (15.9%)			26 (41.3%)		
TAPSE/PASP	Severe (< 0.32)	28 (12.7%)	9 (32.1%)	3.0955	0.0785	26 (92.9%)	19.2880	0.0001*
TAPSE/PASP	Moderate (0.32–0.419)	53 (24.1%)	10 (18.9%)	0.0834	0.7727	40 (75.5%)	12.8381	0.0003*
TAPSE/PASP	Normal (≥ 0.42)	139 (63.2%)	22 (15.8%)			63 (45.3%)		

## Discussion

The present study evaluated echocardiographic predictors of hospital readmission and mortality in elderly patients with HFpEF, incorporating hypertension and diabetes as covariates rather than inclusion criteria. The findings reveal that in a cohort of 220 patients aged ≥60 years admitted with acute decompensated HFpEF, there was a high prevalence of abnormal GLS (62.7%), reduced LA reservoir strain (65.0%), elevated e/e′ ratio (71.4%), increased LA volume index (78.2%), and impaired right ventricular-pulmonary arterial coupling as indicated by low TAPSE/PASP ratio (36.8%). These values are consistent with the high burden of subclinical systolic, diastolic, and right ventricular dysfunction previously documented in elderly HFpEF populations [7].

Baseline demographic and clinical characteristics in the current study align closely with those described in major HFpEF registries. The mean age of 68.4±6.2 years is similar to that reported in the Candesartan in Heart failure: Assessment of Reduction in Mortality and morbidity (CHARM)-Preserved and Irbesartan in Heart Failure with Preserved Systolic Function (I-PRESERVE) sub-analyses, both of which noted mean ages in the late 60s to early 70s [8]. The male predominance (57.3%) in this study differs from the female preponderance typically observed in HFpEF cohorts such as Treatment of Preserved Cardiac Function Heart Failure with an Aldosterone Antagonist (TOPCAT) and Phosphodiesterase-5 Inhibition to Improve Clinical Status and Exercise Capacity in Heart Failure with Preserved Ejection Fraction (RELAX) studies, which may reflect regional differences in risk factor distribution, referral patterns, and healthcare access in the Indian population [9]. The NYHA class distribution, with a majority in Class III (53.6%), indicates advanced functional limitation at admission, comparable to that reported in hospitalized HFpEF patients in the Organized Program to Initiate Lifesaving Treatment in Hospitalized Patients with Heart Failure (OPTIMIZE-HF) registry [10].

The comorbidity profile in the present study - high prevalence of hypertension, diabetes, chronic kidney disease, and coronary artery disease - parallels findings from both Western and Asian HFpEF studies, though the proportion with diabetes (49.5% with elevated HbA1c) is notably higher than in many Western cohorts [11]. This may be due to the higher baseline prevalence of metabolic syndrome and insulin resistance in South Asian populations, a factor that may also accelerate diastolic dysfunction and LA remodeling. The elevated prevalence of atrial fibrillation and chronic obstructive pulmonary disease (COPD) aligns with prior data showing that these comorbidities amplify functional impairment and are linked to higher rehospitalization risk in HFpEF [7,10].

Echocardiographic parameters in this study reveal substantial subclinical systolic dysfunction despite preserved EF, as indicated by impaired GLS in nearly two-thirds of patients. Previous meta-analyses have confirmed that GLS is a more sensitive marker of early myocardial dysfunction than LVEF and that its impairment is common in HFpEF [7]. The prevalence of reduced LA reservoir strain (65%) is also consistent with prior literature demonstrating that LA strain is a sensitive marker of elevated LV filling pressures, diastolic dysfunction, and adverse outcomes [8]. Elevated e/e′ ratio (>15 in 71.4% of patients) and enlarged LA volume index (>34 mL/m² in 78.2%) indicate advanced diastolic dysfunction, mirroring reports from echocardiographic substudy cohorts such as Prospective comparison of ARNI with ARB on Management Of heart failUre with preserved ejectioN fracTion (PARAMOUNT) and Study Assessing the Effect of Atorvastatin on Global Endothelial Function in Heart Failure with Preserved Ejection Fraction (SAGE-HFpEF) [9,10].

Right ventricular-pulmonary vascular uncoupling, assessed by TAPSE/PASP ratio, was abnormal in 36.8% of the present cohort. This finding is important because prior studies have identified right ventricular (RV) dysfunction and elevated pulmonary pressures as strong independent predictors of mortality and HF readmissions in HFpEF [11]. Compared with Western series, the proportion of patients with abnormal TAPSE/PASP in this study is modestly lower, which could reflect earlier detection or under-recognition of advanced pulmonary vascular disease in this demographic. The echocardiographic abnormalities seen here - especially the combination of impaired GLS, low LA strain, high e/e′, and reduced TAPSE/PASP - suggest a multidimensional pathophysiology in elderly HFpEF that involves not only LV diastolic stiffness but also atrial myopathy, RV dysfunction, and pulmonary vascular loading.

The consistency of these findings with global HFpEF data reinforces their generalizability, but the higher prevalence of certain abnormalities (particularly impaired GLS and LA strain) compared to some Western datasets [9-11] may indicate a greater burden of diffuse myocardial fibrosis or microvascular dysfunction in the South Asian elderly population. This could possibly be related to higher cumulative exposure to hypertension and diabetes from an earlier age, dietary factors, and genetic susceptibility to small-vessel disease. 

The prognostic role of right ventricular-pulmonary vascular coupling, as measured by the TAPSE/PASP ratio, is increasingly recognized in HFpEF, particularly in elderly inpatients with multiple comorbidities. In the present study, a TAPSE/PASP ratio below 0.42 was strongly associated with readmission, with the steepest gradient observed in those with severe uncoupling (<0.32), who experienced 32.1% mortality and nearly 93% readmission within a year. These results align with findings from prior cohort analyses, which have demonstrated that impaired RV-PA coupling reflects the combined burden of pulmonary hypertension and right ventricular dysfunction, both of which independently worsen prognosis in HFpEF patients [12]. The pathophysiological mechanism likely involves progressive pulmonary vascular remodeling secondary to chronic left-sided congestion, leading to increased afterload on the right ventricle and eventual RV failure. This maladaptive process is exacerbated in elderly patients with long-standing hypertension, atrial fibrillation, or chronic lung disease, all of which were prevalent in our cohort.

The interplay between comorbidities and echocardiographic prognosticators is a critical consideration in real-world HFpEF management. In our cohort, poorly controlled diabetes, chronic kidney disease, and anemia were each associated with a higher prevalence of abnormal GLS, elevated e/e′, and reduced LA strain. This is consistent with earlier studies showing that metabolic and renal comorbidities accelerate myocardial fibrosis, increase chamber stiffness, and impair both systolic and diastolic reserve [13,14]. These systemic conditions may also attenuate the prognostic value of a single echocardiographic parameter, reinforcing the need for a multidimensional approach when risk stratifying elderly HFpEF patients. For instance, while reduced GLS was predictive of adverse outcomes across the overall population, its specificity for cardiovascular death was lower in patients with concomitant chronic kidney disease, likely due to competing non-cardiac mortality risks.

From a clinical management perspective, our results highlight the importance of incorporating these advanced echocardiographic measures into inpatient and early post-discharge planning. Patients identified with severely impaired GLS, markedly reduced LA strain, or TAPSE/PASP uncoupling should be targeted for more aggressive diuretic titration, closer outpatient follow-up, and early referral to multidisciplinary HF clinics. Prior evidence has demonstrated that early, structured follow-up after HF hospitalization can reduce readmissions and improve quality of life, particularly when combined with targeted management of comorbidities [15]. In our cohort, high-risk echocardiographic profiles frequently coexisted with elevated NT-proBNP and clinical congestion despite apparent hemodynamic stability, suggesting that discharge readiness assessments should integrate both biochemical and imaging criteria.

These findings also carry implications for alignment with guideline-directed management. While current ESC and ACC/AHA guidelines emphasize the use of natriuretic peptides and standard echocardiographic measures for diagnosis, our data suggest that integrating GLS, LA reservoir strain, and TAPSE/PASP ratio could refine prognostic classification in hospitalized elderly HFpEF patients [16]. Such an integration may facilitate more personalized therapy, guiding decisions about the intensity of diuresis, initiation of sodium-glucose cotransporter-2 inhibitors (SGLT2) inhibitors, or evaluation for pulmonary hypertension-specific therapies in select patients. Furthermore, given the growing body of literature supporting the prognostic role of these parameters, incorporation into standardized HFpEF risk scores should be considered in future updates to international guidelines.

Strengths of the study

The primary strength of this study lies in its prospective design with systematic recruitment of a well-defined elderly HFpEF cohort over a one-year period, ensuring temporal consistency in data collection. All echocardiographic measurements, including advanced parameters such as GLS, LA reservoir strain, and TAPSE/PASP ratio, were obtained following standardized ASE guidelines and interpreted by blinded cardiologists, minimizing observer bias. The relatively large sample size of 220 patients for a single-center HFpEF study enhances the statistical power to detect meaningful associations between echocardiographic parameters and clinical outcomes. Additionally, the structured follow-up at multiple time points up to one year allowed for a detailed temporal assessment of both mortality and readmissions, providing a comprehensive outcome profile. The integration of both conventional and advanced echocardiographic indices, alongside clinical and biochemical variables, strengthens the study’s translational relevance and applicability in real-world settings.

Limitations of the study

Despite its strengths, the study has certain limitations. Being a single-center study conducted in a tertiary referral hospital, the findings may not be fully generalizable to community-based HFpEF populations or to younger cohorts. As an observational study, causal relationships cannot be firmly established, and unmeasured confounders may have influenced the associations observed. Advanced echocardiographic techniques such as speckle-tracking require high-quality imaging, leading to the exclusion of patients with suboptimal images, which could have introduced selection bias. The external validation and comparison with multicenter cohorts were not performed, which limit the generalizability of our findings. The present analysis was primarily based on univariate associations, and multivariate adjustment for all potential confounders was not feasible given the event rates, which may have led to residual bias in effect size estimates. Subgroup analyses (e.g., by sex, comorbidities, or atrial fibrillation status) were not performed, which limits our ability to determine whether prognostic associations differed across clinically relevant subpopulations. Combined abnormalities, such as the coexistence of renal dysfunction and anemia, were not specifically analyzed, which may underestimate the contribution of multimorbidity burden to adverse outcomes. Additionally, while echocardiographic parameters were analyzed as predictors, the study did not evaluate whether interventions guided by these parameters could improve outcomes, limiting direct clinical application. Biomarkers other than NT-proBNP, which could have provided further mechanistic insights, were not assessed. We did not perform additional stratification of echocardiographic parameters by NYHA class, nor did we analyze the overlap of TAPSE and TAPSE/PASP abnormalities, which may limit subgroup interpretation. Atrial fibrillation at baseline limited the feasibility of LA strain and e/e′ measurements in some patients, which was a methodological limitation. Serial echocardiographic reassessment during follow-up was not performed, and this represents an important area for future research. Finally, the study’s follow-up was limited to one year, and longer-term prognostic implications of these echocardiographic markers remain to be established.

## Conclusions

This study highlights the prognostic role of advanced echocardiographic indices in elderly patients with HFpEF. Despite preserved ejection fraction, many exhibited subclinical systolic dysfunction, atrial impairment, elevated filling pressures, and RV-PA uncoupling, all linked to adverse outcomes. Impaired GLS, reduced LA reservoir strain, elevated e/e′, and low TAPSE/PASP consistently predicted readmission, with severity grading showing a clear gradient of risk. Comorbidities such as hypertension, diabetes, renal disease, and coronary artery disease further amplified the impact of these abnormalities. Clinically, these findings support incorporating myocardial strain, atrial strain, and RV-PA coupling into HFpEF assessment. They are feasible, reproducible, and add prognostic information beyond traditional parameters, helping identify high-risk patients for closer follow-up and tailored care. The high readmission and mortality rates observed reflect global HFpEF trends, emphasizing the need for proactive, multidisciplinary strategies. Detailed echocardiographic evaluation thus offers not only diagnostic but also prognostic utility in guiding elderly HFpEF management.
